# Leaf open time sinogram (LOTS): a novel approach for patient specific quality assurance of total marrow irradiation

**DOI:** 10.1186/s13014-020-01669-2

**Published:** 2020-10-14

**Authors:** Rajesh Thiyagarajan, Dayananda Shamurailatpam Sharma, Suryakant Kaushik, Mayur Sawant, K. Ganapathy, N. Arunai Nambi Raj, Srinivas Chilukuri, Sham C. Sundar, Kartikeswar Ch. Patro, Arjunan Manikandan, M. P. Noufal, Rangasamy Sivaraman, Jose Easow, Rakesh Jalali

**Affiliations:** 1 Department of Medical Physics, Apollo Proton Cancer Centre, 100 Feet Road Tharamani, Chennai, 600096 Tamil Nadu India; 2 Department of Radiation Oncology, Apollo Proton Cancer Centre, 100 Feet Road Tharamani, Chennai, 600096 Tamil Nadu India; 3 Centre for Biomaterials, Cellular and Molecular Theranostics (CBCMT), VIT University, Vellore, 632014 India; 4 School of Advanced Sciences, VIT University, Vellore, 632014 India; 5 Department of Haematology, Blood and Marrow Transplantation, Apollo Speciality Hospital, Teynampet, Chennai India

**Keywords:** Sinogram, Exit dosimetry, Dose Reconstruction, Patient specific QA, Total marrow irradiation, Helical tomotherapy, MVCT

## Abstract

There is no ideal detector-phantom combination to perform patient specific quality assurance (PSQA) for Total Marrow (TMI) and Lymphoid (TMLI) Irradiation plan. In this study, 3D dose reconstruction using mega voltage computed tomography detectors measured Leaf Open Time Sinogram (LOTS) was investigated for PSQA of TMI/TMLI patients in helical tomotherapy. The feasibility of this method was first validated for ten non-TMI/TMLI patients, by comparing reconstructed dose with (a) ion-chamber (IC) and helical detector array (ArcCheck) measurement and (b) planned dose distribution using 3Dγ analysis for 3%@3mm and dose to 98% (D_98%_) and 2% (D_2%_) of PTVs. Same comparison was extended for ten treatment plans from five TMI/TMLI patients. In all non-TMI/TMLI patients, reconstructed absolute dose was within ± 1.80% of planned and IC measurement. The planned dose distribution agreed with reconstructed and ArcCheck measured dose with mean (SD) 3Dγ of 98.70% (1.57%) and 2Dγ of 99.48% (0.81%). The deviation in D_98%_ and D_2%_ were within 1.71% and 4.10% respectively. In all 25 measurement locations from TMI/TMLI patients, planned and IC measured absolute dose agreed within ± 1.20%. Although sectorial fluence verification using ArcCHECK measurement for PTVs chest from the five upper body TMI/TMLI plans showed mean ± SD 2Dγ of 97.82% ± 1.27%, the reconstruction method resulted poor mean (SD) 3Dγ of 92.00% (± 5.83%), 64.80% (± 28.28%), 69.20% (± 30.46%), 60.80% (± 19.37%) and 73.2% (± 20.36%) for PTVs brain, chest, torso, limb and upper body respectively. The corresponding deviation in median D_98%_ and D_2%_ of all PTVs were < 3.80% and 9.50%. Re-optimization of all upper body TMI/TMLI plans with new pitch and modulation factor of 0.3 and 3 leads significant improvement with 3Dγ of 100% for all PTVs and median D_98%_ and D_2%_ < 1.6%. LOTS based PSQA for TMI/TMLI is accurate, robust and efficient. A field width, pitch and modulation factor of 5 cm, 0.3 and 3 for upper body TMI/TMLI plan is suggested for better dosimetric outcome and PSQA results.

## Introduction

Total body irradiation (TBI) is integral to myeloablative conditioning (MAC) regimen in patients requiring allogeneic bone marrow transplant (ABMT) for myeloid and lymphoid leukemia. The conditioning regimens incorporating TBI have shown better clinical outcome compared to chemotherapy alone [1–3]. However, the practice of TBI has witnessed a steady decline as a result of radiation induced toxicity and alternative use of only chemotherapy based conditioning regimens. Profoundly, several investigators have reported the feasibility of selective irradiation of Total Marrow (TMI) and Lymphatic (TMLI), using volumetric modulated arc therapy (VMAT) and more conveniently with helical tomotherapy (HT) [4–7]. TMI have shown a significant reduction of dose to organs at risk (OAR) as compared to TBI, thereby reducing radiation induced toxicity [5, 6, 8, 9] with encouraging complete response rate [9].

The treatment planning of TMI/TMLI using either HT or VMAT is complicated due to extensive and complex irregular shape target volume requiring highly modulated beam intensity, multiple isocenters and field junctions. HT has been the most preferred high precision treatment modality for TMI/TMLI owing to its ability to treat a maximum length of 135 cm at a time, requiring zero or one junction to treat the entire target volume as compared to 4–5 junctions in VMAT [4–7]. Pre-treatment verification of dose delivery in the entire target volume is of paramount importance to ensure accurate delivery of dose to the patient. Conversely, verification of TMI/TMLI treatment plan poses many challenges due to the non-availability of suitable detectors or measuring equipment. All the commercially available active or passive detectors for patient specific quality assurance (PSQA) have limited longitudinal and lateral dimensions of 20–25 cm to verify highly modulated mega treatment fields from TMI/TMLI especially for HT delivery. The feasibility of different detector arrays (2D or 3D) arranged in planar, hexagonal, circular and helical have been investigated for section by section dose verification of TBI/TMI plans [7, 10, 11]. Takahashi and Hui [12] developed a simple in-house whole body phantom to verify TMI treatment plan of up to 110 cm in HT using three ionization chambers and three radiochromic films. However, all the investigated detectors and methodologies were complex, unable to measure complete fluence in a single set-up and lack in efficiency.

The unique design of megavoltage cone beam computed tomography (MVCT) detectors in HT allows measurement of delivered sinogram (dose fluence) prior to or during treatment. Several studies have reported the feasibility of using measured sinogram to reconstruct 3D dose distribution on to the patient CT datasets using in-build or independent dose reconstruction algorithm in routine clinical cases [13–19]. To the best of our knowledge, no study has been conducted to provide an accurate and yet efficient PSQA of TBI/TMI/TMLI patients treated on HT. In this study, a 3D dose reconstruction method from MVCT detectors measured leaf open time sinogram (LOTS) was validated for PSQA in a routine clinical environment. The same PSQA method was investigated to assess the delivery accuracy of TMI/TMLI treatment plans. The sensitivity of this PSQA method on HT planning parameter was also investigated for TMI/TMLI patients.

## Materials and methods

### Treatment unit

Radixact X9 used in this study is the latest generation MVCT image guided HT system supplied by Accuray, Inc., Sunnyvale, USA. Although system configuration remains almost the same as its predecessor, the gantry has been redesign to incorporate kV X-ray based imaging and real-time motion management functionality. However, our model was installed prior to the commercial release of synchrony motion management functionality. The dose rate from the 6 MV flattening filter free (FFF) beam has increased to 1000 MU/min. Intensity modulation is still achieved using 64 binary multi-leaf collimator (MLC), each of 6.25 mm projected leaf width at isocenter. The user selectable field width along the longitudinal direction remains at 1, 2.5 and 5 cm, whereas maximum lateral dimension is 40 cm. However, the jaws positions along longitudinal direction can be optimize dynamically to achieve better dose conformity in superior and inferior end of the target. A couch catcher assembly installed on the rear side of the gantry supports couch during treatment and reduces the couch sag. The gantry rotation periods remains between 1 to 5 rotation per minute (RPM).

### MVCT detector

The MVCT detector in the Radixact HT system is an arc-shaped detector located opposite to the accelerator on the ring gantry. It consists of an array of 640 channels, each with two parallel plate ionization cavities filled with pressurized Xenon gas under 5 atmospheric pressure. The dimension of each detector channel is 1.24 mm in the transverse and 25.4 mm in longitudinal direction. The source to detector distance is 145 cm. The imaging field of view (FOV) defined by the width of the MLC is 39.4 cm at isocenter. Therefore, of the 640 detector channels, 576 are connected to the data acquisition system and only 520 are used in MVCT.

### 3D dose reconstruction from MLC-LOTS

A unique feature of Radixact HT and Delivery Analysis software (v1.2.2.7, Accuray, Inc., Sunnyvale, USA) is its ability to analyze the exit/transmitted radiation fluence recorded by the in-build MVCT detectors. During the irradiation of a treatment plan in the absence of any object on the TomoCouch, the detector sinogram plot was constructed from the signal pulses collected by every three central detector channels corresponding to each MLC leaf. These signal pulses were segmented into projection bins using treatment plan information. In each projection, the pulse value above the set threshold was used for the calculation of LOT. The MLC-LOTS recorded and reconstructed after the end of each irradiation were retrospectively used to reconstruct the dose distribution on the same patient CT dataset in the Delivery Analysis workstation. Readers may refer publications by Kapatoes et al. [13, 14] for detail theoretical explanation and proof of concept.

### TMI/TMLI treatment planning

Treatment plans (3 TMI and 2 TMLI) of five adult patients treated during April 2019 till Feb 2020 were used for this retrospective study. Briefly, for each patient, HT treatment plan was generated in Precision (V 2.1.1.1, Accuray, Inc., Sunnyvale, USA) treatment planning system (TPS), separately for the upper and lower body. Planning parameters, shown in Table 1, were chosen as a balance between plan quality, delivery efficiency and also in alignment with previous publications [4, 7, 8]. Every plan was optimized to deliver a homogeneous dose of 12 Gy in 6 fractions to the entire target volume. The dose heterogeneity in the junction region of the upper and lower body was maintained within ± 5% of the prescription dose. The optimization engine of the TPS iteratively modify the leaf open time (LOT) of the MLC from different projection angle to closely achieve the defined planning clinical goals. The final dose calculation eliminates LOT less than 20 ms to minimize the leaf latency related dosimetric error [20]. The resultant LOT of different leaves at different gantry positions were represented as planned sinogram. The outcome of every HT treatment plan were evaluated thoroughly using standard dose volume indices for the targets and OARs and fulfill our pre-defined clinical goals. The detail report on treatment simulation and planning is beyond the scope of this study.

**Table 1:** Helical tomotherapy treatment plan parameters of Non-TMI, TMI and TMLI patients

Patient	Diagnosis	Treatment plan parameter		
		Field width (cm)	Pitch	Modulation factor
Non-TMI patients				
P1	Anaplastic oligodendroglioma	2.5	0.43	2.20
P2	Glioblastoma	1.0	0.43	2.00
P3	Glioblastoma	1.0	0.41	2.20
P4	Ca head of pancreas	1.0	0.41	2.18
P5	Oligometastatic Ca Lung	1.0	0.43	2.35
P6	Ca tongue recurrent	1.0	0.41	2.35
P7	Ca right breast	2.5	0.28	2.40
P8	Ca rectum post op	2.5	0.43	2.00
P9	Ca esophagus	2.5	0.30	2.10
P10	Meningioma	1.0	0.41	1.90
TMI/TMLI patients				
P11-HFS	Chronic myloid leukemia	5.0	0.31	3.50
P11-FFS		5.0	0.40	2.50
P12-HFS	Acute lymphoblastic leukemia	5.0	0.41	2.80
P12-FFS		5.0	0.43	2.00
P13-HFS	Acute lymphoblastic leukemia	5.0	0.31	3.50
TP13-FFS		5.0	0.41	2.40
P14-HFS	Chronic myloid leukemia	5.0	0.30	3.00
P14-FFS		5.0	0.40	2.40
P15-HFS	Acute lymphoblastic leukemia	5.0	0.43	2.49
P15-FFS		5.0	0.40	2.15

### Validation of 3D dose reconstruction and analysis

The feasibility and accuracy of Delivery Analysis reconstructed 3D dose distribution were first validated for ten non-TMI patients, representing a wide spectrum of clinical sites and level of complexities in treatment plan parameters such as field width, modulation factor and pitch as summarized in Table 1. For each clinically approved treatment plan, pre-treatment PSQA was carried out following departmental protocol of absolute point dose measurement in Cheese phantom (Accuray, Inc., Sunnyvale, USA) using calibrated 0.053 cc Extradin A1SL (Standard Imaging, Inc. Middleton, WI) ionization chamber (IC) and 2D dose fluence measurement using ArcCHECK (SunNuclear, Suntree Blvd Melbourne) helical diode arrays. ArcCHECK is a cylindrical PMMA phantom of 26.6 cm diameter having 1386 (0.016 mm^3^) diodes arranged in a helical manner, providing detector spacing of 10 mm and covering a treatment length (cranio-caudal) of 20 cm. The measured fluence at the detector plane was unwrapped using ArcCHECK software and compared against the Precision TPS calculated fluence using 2D gamma (γ) analysis set at 3% dose difference at 3 mm distance-to-agreement (3%@3mm). The PSQA results were considered acceptable if 95% of the total number of analyzed pixels have γ value less than one.

In addition, the same plans were delivered on Radixact HT without any object on the carbon-fiber flat Tomo couch. The MVCT detector measured LOTS was used to reconstruct the 3D dose distribution on to the CT datasets of the patient in Delivery Analysis for verification of absolute point dose and 3D dose distribution either by using 3D gamma analysis or standard dose-volume-histogram (DVH). The same gamma acceptance criteria of 3%@3mm were used for 3Dγ analysis. Whereas, dose to 98% (D_98%_) and 2% (D_2%_) of planning target volume (PTV) extracted from the cumulative DVHs of planned and reconstructed dose distribution were compared to evaluate agreement in minimum and maximum dose to target.

The potential application of the MLC-LOTS for PSQA was extended for the ten treatment plans (two per patient) of the five previously treated TMI and TMLI patients. For every patient, PSQA plans were created for each of the two clinically approved treatment plans (one each for upper and lower body) by recalculating the dose distribution on a standard cheese phantom. This was required to enable the clinically approved treatment plan to be delivered on Radixact HT in QA mode. However, these plans were delivered on Radixact HT without any phantom. The MVCT detector recorded transmitted radiation from the carbon-fiber Tomo couch were used to reconstruct the MLC-LOTS, which subsequently was used to reconstruct the 3D dose distribution on the CT datasets of the patient. Accuracy of the delivered dose fluence was verified by comparing (a) precision TPS calculated (planned) versus IC measured absolute dose in Cheese phantom, (b) planned versus reconstructed 3D dose distribution from measured LOTS using 3D γ analysis and (c) planned versus reconstructed 3D dose distribution from measured LOTS using standard dose volume histogram (DVH) of D_98%_ and D_2%_. Based on the verification results, all the five upper body TMI/TMLI plans were re-optimized using fixed field width of 5 cm, pitch of 0.43 and modulation factor same as in the original plans. To further investigate, the influence of HT planning parameter on the LOTS based PSQA results, another five plans of the upper body TMI/TMLI were created with a field width of 5 cm and pitch of 0.3 and modulation factor of 3. All the ten new plans were optimized such that the final dose distribution were comparable or better than the clinically delivered plans. For the ten new upper body TMI/TMLI plans, PSQA based on 3D dose reconstruction from MVCT measured MLC-LOTS were carried out as described above.

## Results

The comparison of planned, measured and reconstructed absolute point dose, 2Dγ and 3Dγ, D_98%_ and D_2%_ of PTVs of ten non-TMI patients are presented in Table 2. Off the ten clinical plans, planned and ion chamber measured absolute dose agrees within ± 1% in eight and 1.80% in two plans with overall mean (SD) of 0.1% (0.89%). Whereas, the agreement between reconstructed and IC measured absolute dose was within ± 1% in six and ± 1.8% in four plans, resulting in overall mean (SD) of 0.14% (1.03%). In eight patients, planned dose agrees within ± 1% with that of reconstructed dose while in two patients the agreement was within ± 1.2%. In all the plans, both 2Dγ and 3Dγ were above 96% with an overall mean (SD) of 99.48% (0.81%) for planar dose comparison between planned and ArcCHECK measurement; and 98.7% (1.57%) for volumetric dose comparison between planned and reconstructed dose distribution respectively. The planned and reconstructed D_98%_ and D_2%_ to PTVs for the ten non-TMI patients agreed with maximum (mean ± SD) of 1.71% (0.47% ± 0.9%) and 4.1% (1.42% ± 1.39%) respectively.

**Table 2:** Comparision of absolute point dose, 2D and 3D gamma, absolute dose volume (D_99%_ and D_2%_ to PTV) amongst TPS calculated (planned), ion chamber measured and reconstructed dose distribution from LOTS for ten non-TMI patients treated for various clinical sites on RadiXact HT

Non-TMI Patient	Absolute dose (cGy) on cheese phantom			2D/3D gamma (γ%) values between TPS calculated and		Deviation (%) between reconstructed and TPS calculated dose	
	Planned	Ion chamber measured	Reconstructed from LOTS	ArcCheck measured 2Dγ%	Reconstructed from LOTS 3Dγ%	D_98%_ of PTV	D_2%_ of PTV
P1	139	137.84	140	99.4	97	0.56	3.01
P2	135	134.88	135	99.8	100	0.11	0.96
P3	141	140.2	141	100	97	1.71	4.10
P4	172	171.51	171	100	100	− 0.31	− 0.15
P5	167	166.67	166	99.9	100	− 0.61	− 0.49
P6	144	143.15	145	100	96	0.28	1.47
P7	198	201.12	200	99.5	98	1.65	2.03
P8	176	175.72	178	100	99	− 0.26	1.63
P9	149	149.65	149	98.7	100	− 0.09	1.09
P10	122	124.13	122	97.5	100	1.70	0.57

**Fig. 1:**
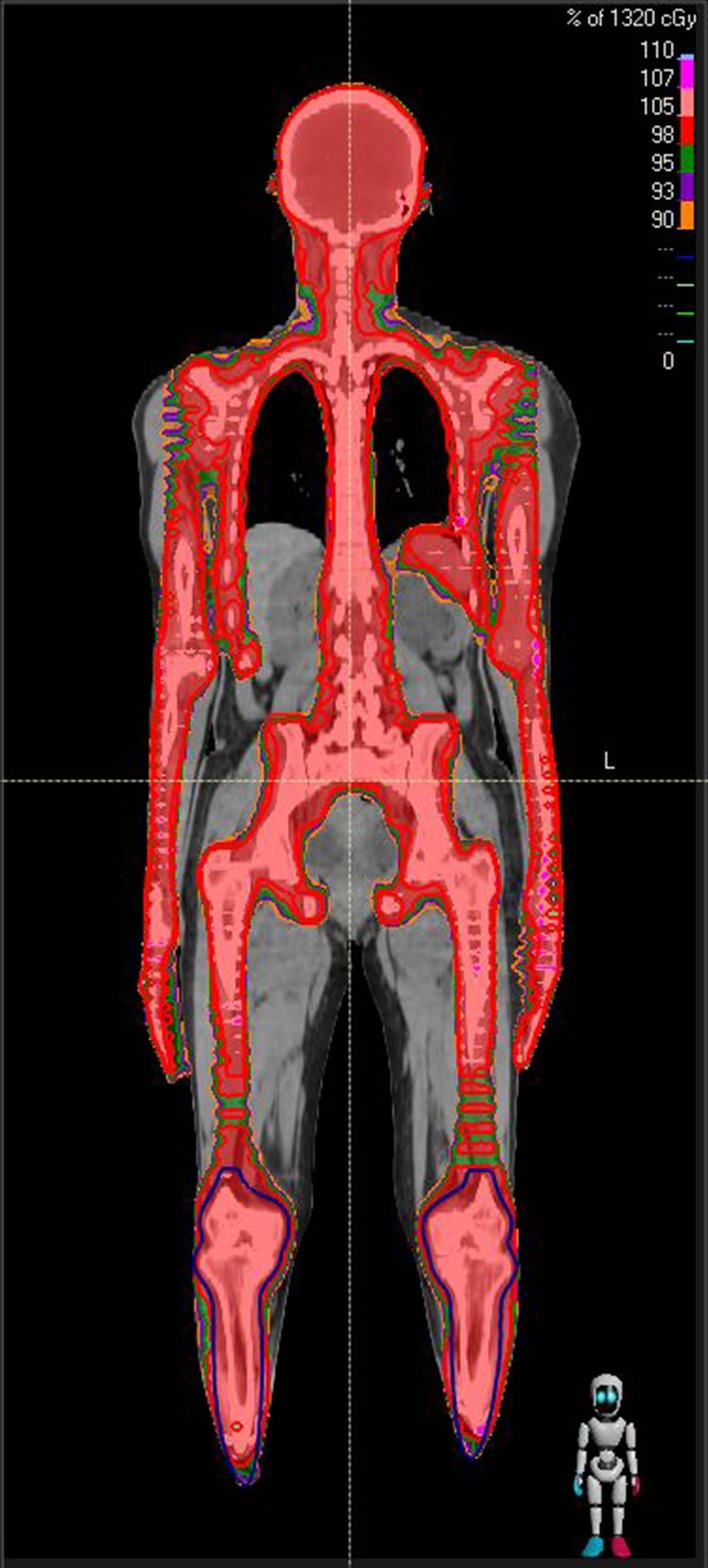
Composite dose distribution from upper and lower body plans of a representative TMI patients showing selective irradiation of total marrow

**Fig. 2:**
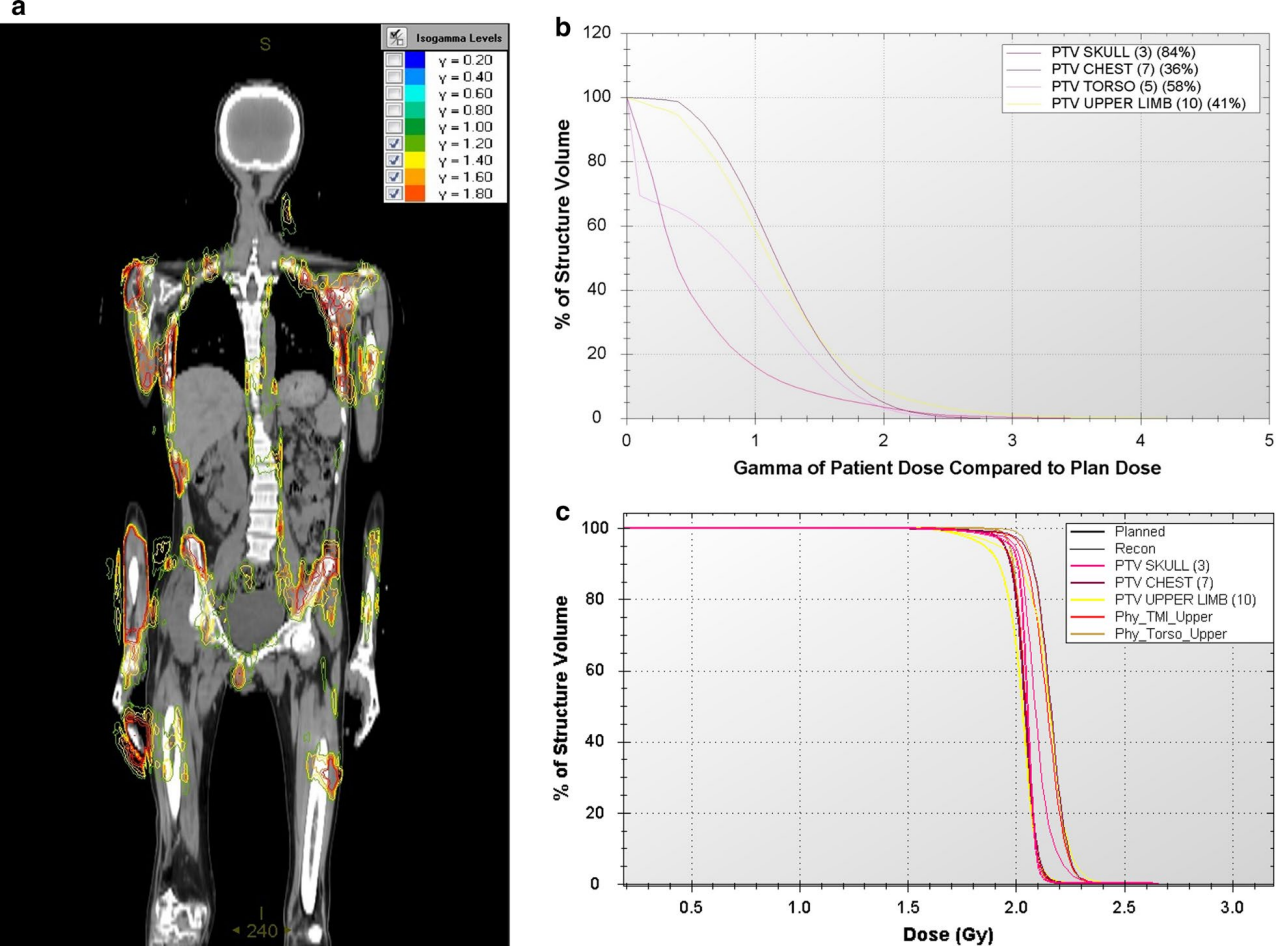
**a** Isogamma levels greater than one resulted from the comparison of planned and reconstructed dose distribution from measured LOTS for one of the representative upper body TMI treatment plan. 3Dγ was analyse using 3% dose difference at 3 mm distance to agreement. **b** 3Dγ plots of four separate planning target volumes (PTVs) in upper body TMI plan of a representative patient. **c** Comparison of planned and reconstructed cumulative dose volume histogram (DVH) of four separate planning target volumes (PTVs) in upper body TMI plan

**Fig. 3:**
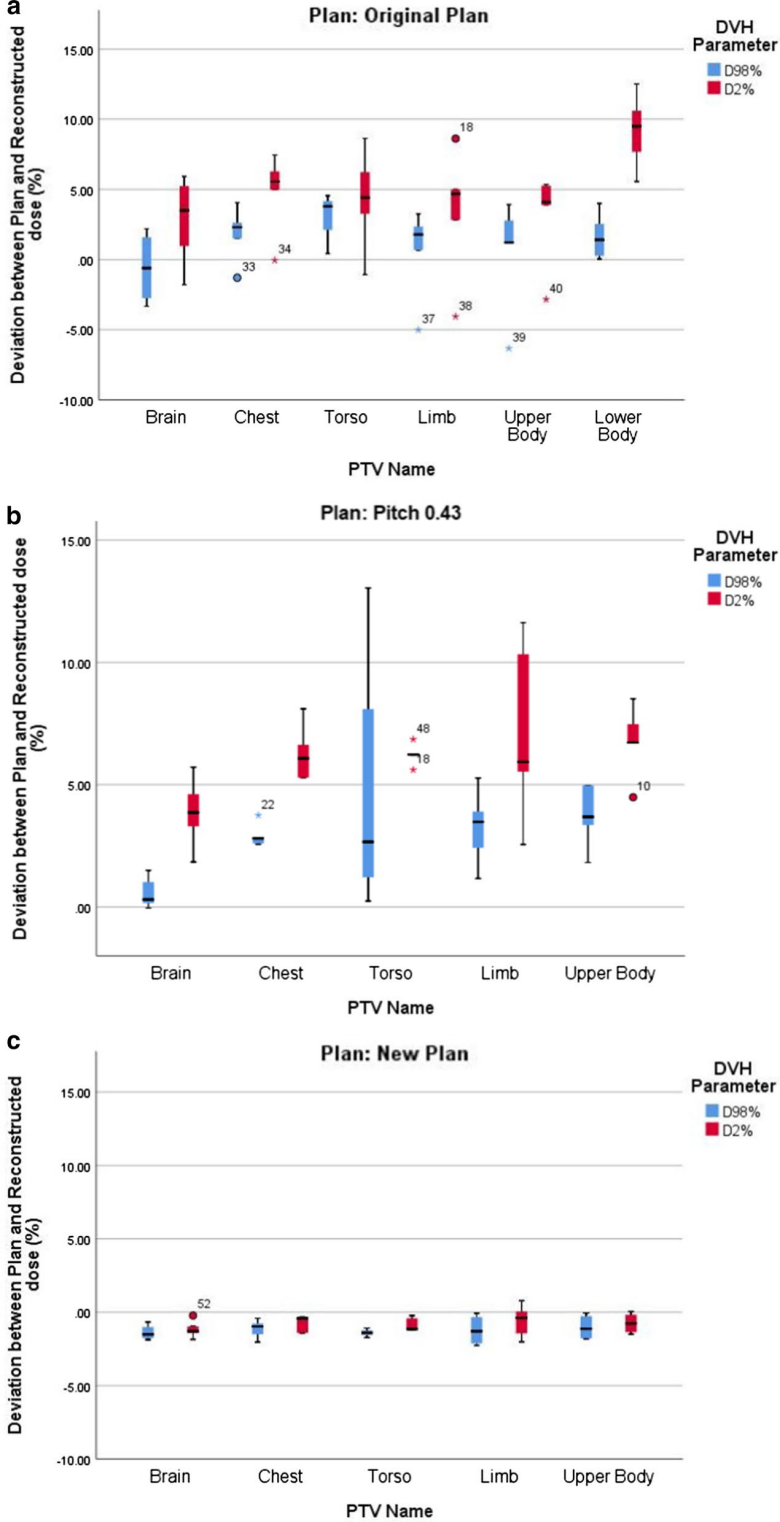
**a** Deviation between planned and reconstructed D_98%_ and D_2%_ of various PTVs from the (**a**) original TMI/TMLI plans. **b** new TMI/TMLI plans with new pitch of 0.43 and modulation factor same as original plans. **c** new TMI/TMLI plans with new pitch of 0.3 and modulation factor of 3

The composite dose distribution of upper and lower body treatment plan for a representative TMI patient is shown in Fig. 1. It showed selective irradiation of marrow with the prescribed dose uniformly while sparing the surrounding normal tissues including the island OARs such as lungs, bowels, prostates, etc. The detail analysis of dosimetric outcome is beyond the scope of this study. The comparison of planned and ion chamber measured absolute doses at five representative locations of each TMI/TMLI plans are summarized in Table 3. In all 25 locations, planned and IC measured absolute dose agreed within ± 1.2% (mean = 0.04%, SD = 0.55%). However, large disagreement was observed between planned and reconstructed 3D dose distribution with many voxels having dose difference more than 3% at 3 mm in upper body plan as represented by isogamma levels greater than one in Fig. 2a for a representative patient. It was also evident from the corresponding 3Dγ plots (Fig. 2b) and comparative DVHs (Fig. 2c) of various PTVs, that planned and reconstructed 3D dose distribution does not agree well both in terms of 3Dγ and values of D_98%_ and D_2%_. Table 4 represents the 3Dγ of various PTVs resulted from the comparison of planned and reconstructed dose distribution of the five TMI/TMLI patients. None of the upper body plans pass 3Dγ criteria of 95% to all PTVs, although it was comparatively better for patients P13 and P14. The mean (SD) 3Dγ for PTVs brain, chest, torso, limb, combination of all PTVs in upper body, external of upper body and lower body were 92.00% (5.83%), 64.80% (28.28%), 69.20% (30.46%), 60.80% (19.37%), 73.2% (20.36%) 84.00% (11.98%) and 100% (0%) respectively. However, all the lower body plans showed excellent agreement with mean 3Dγ of 100% for PTV lower body and external body. Figure 3a shows the deviation between planned and reconstructed D_98%_ and D_2%_ of various PTVs. The median deviation in D_98%_ of various PTVs were − 0.60, 2.30, 3.79, 1.78, 1.20 and 1.39 for brain, chest, torso, limb, upper body and lower body respectively. The corresponding deviations in D_2%_ were larger in all PTVs with 2.22, 5.56, 6.23, 4.68, 4.09 and 9.48 respectively.

**Table 3:** Comparison of planned and ionization chamber measured point dose at five representative points of five anatomical sites of each TMI/TMLI patient

Site	P11		P12		P13		P14		P15	
	Planned (cGy)	Measured (cGy)	Planned (cGy)	Measured (cGy)	Planned (cGy)	Measured (cGy)	Planned (cGy)	Measured (cGy)	Planned (cGy)	Measured (cGy)
Brain	164	163.91	165	166.47	162	163.12	164	162.09	186	185.66
Chest	177	177.02	193	193.86	198	198.87	169	168.5	184	182.69
Pelvis	191	190.77	205	205.31	201	202.23	174	173.5	208	210.34
Upper leg	149	149.34	172	172.54	151	151.33	152	151.69	169	167.27
Lower leg	141	141.26	153	153.42	149	149.48	149	148.03	186	185.77

**Table 4:** 3D gamma (γ%) values resulted from the comparison of planned and reconstructed dose distribution in 6 planning target volumes (PTVs) and two external (body) from the two plans (upper body and lower body) of every TMI/TMLI patients. 3Dγ values of 3%@3mm from the original plans were obtained with different pitch and modulation factor listed in Table 1, whereas 3Dγ values of 3%@3mm with pich modified to 0.43 from the original plans were also shown here. The 3Dγ values with 2%@2mm from another set of new plans of the same patients with fixed pitch of 0.3 and modulation factor of 3 showed significant improvement in the 3Dγ values

Treatment plan	Target (PTV)	3Dγ (3%@3mm) from original plans					3Dγ (3%@3mm) from new plans with field width = 5 cm; pitch = 0.43 and modulation factor = same as original plans					3Dγ (2%@2mm) from new plans with field width = 5 cm; pitch = 0.3 and modulation Factor = 3				
		P11	P12	P13	P14	P15	P11	P12	P13	P14	P15	P11	P12	P13	P14	P15
HFS	Brain	90	83	97	97	93	72	96	95	92	99	100	94	97	97	100
	Chest	75	28	86	94	41	53	57	68	63	60	100	99	94	100	100
	Torso	83	52	94	93	24	60	68	82	51	46	100	98	100	100	100
	Limb	85	34	73	55	57	82	50	55	51	44	100	95	97	100	100
	Upper body	86	50	91	87	52	67	69	78	63	63	100	97	97	98	100
	External upper body	94	68	95	88	75	87	79	88	82	77	100	99	99	100	100
FFS	Lower body	100	100	100	100	100										
	External lower body	100	100	100	100	100

As a part of the TMI/TMLI implementation protocol in our Institute, pre-treatment planar dose verification of every patient was carried out only for chest PTVs by comparing planned and ArcCHECK measured dose distribution. All patients showed 2Dγ (3%@3mm) above 96% with a mean ± SD of 97.82% ± 1.27% as compared to 3Dγ of 64.80% ± 28.28% from reconstruction method. The poor disagreement between 2Dγ and 3Dγ of the upper body TMI/TMLI plans lead us to investigate the influence of HT plan parameters on the 3Dγ passing rate. First, all the five upper body original TMI/TMLI plans were re-optimize by changing the pitch to 0.43 while retaining the original modulation factor (2.49 to 3.5) and field width. Secondly, all the five original upper body TMI/TMLI plans were re-optimized using fix field width of 5 cm, pitch and modulation factor of 0.3 and 3 respectively. The ten new plans thus created were dosimetrically comparable or slightly better as compare to clinically delivered (original) plans. Analysis of MLC-LOTS of clinically delivered plans and new plans with pitch of 0.3 and modulation factor of 3 lead to the reduction of maximum LOT from 15.06 to 21.41% in four of five plans with reduced standard deviation (SD) ranging from 0.73 to 27.26% in all plans. The mean LOT were also reduced ranging from 9.82 to 31.29% in three patients while it was increase by around 3% in two plans. The average reduction of maximum and mean LOT for the five new plans were 14.95% and 14.86% respectively. In contrast, the change of pitch to 0.43 without changing the modulation factor and field width, lead to an overall increase in the average value of maximum and mean LOT to 29.48% and 18.25% respectively. All the ten newly generated plans were exposed to Radixact HT as described before and measured LOTS were used to reconstruct the 3D dose distribution on to the same patient CT datasets. In all five new upper body plans with new pitch of 0.43, 3Dγ (3%@3mm) were less than the acceptable criteria of 95% for majority of the PTVs. Correspondingly, the agreement of planned and reconstructed values of D_98%_ and D_2%_ showed large deviation as shown in Fig. 3b. However, for the other five new plans with pitch of 0.3 and modulation factor of 3, planned and reconstructed dose agrees with 3Dγ (3%@3mm) values of 100% for all PTVs and external bodies. The agreement even at 2%@2mm criteria were ≥ 94% in all plans and for all PTVs with mean (SD) of 97.6% (2.51%), 98.6% (2.61%), 99.6% (0.89%), 98.4% (2.3%), 98.4% (1.52%) and 99.6% (0.55%) for brain, chest, torso, limb, upper body and external respectively (Table 4). This also leads to significant improvement in the agreement of planned and reconstructed values of D_98%_ (*p* = 0.001) and D_2%_ (*p* = 0.001) for all PTVs in all plans (Fig. 3c). The median deviation in D_98%_ (D_2%_) of PTVs brain, chest, torso, limb and upper body were − 1.52% (− 1.37%), − 0.98% (− 0.42%), − 1.40% (− 1.21%), − 1.30% (− 0.92%), − 1.13% (− 1.07%) respectively.

## Discussion

Our current standard protocol for pre-treatment PSQA of TMI/TMLI patient treatment on Radixact HT include verification of absolute dose in five locations corresponding to brain, chest, pelvis, upper leg and lower leg using calibrated ionization chamber inserted in Cheese phantom and 2D fluence verification only for the chest target using ArcCHECK helical detector array. ArcCHECK allows measurement of treatment field length ≤ 20 cm in a single irradiation. Our protocol is in alignment with other studies wherein several authors have reported verification of absolute dose and fluence either section by section or in the junction of two consecutive arcs using various detectors and methods [7, 11]. In addition, for each patient we also carried out in-vivo EBT3 film dosimetry (a) at eleven pre-defined locations across the whole body and (b) in the junction region of upper and lower body treatment plans to ensure delivery of homogeneous dose. The excellent agreement between planned and measured absolute dose (< ± 1.2%) and planar dose fluence (2Dγ > 96%) in the five TMI/TMLI patients were within the internationally acceptable criteria. Off the many sub PTVs, we have limited the fluence verification only for the PTV chest due to logical and technical challenges prior to treatment and postulated that the results may be still applicable to other PTVs. We have chosen PTV chest because of its complicated shape with many OARs located as an island and hence represent the most complex intensity fluence. The probable shortcoming of our protocol is extensive time spend for the delivery QA, verification of limited dose fluence of a mega treatment volume, challenges in the in-vivo absolute dose verification in a highly modulated heterogeneous dose region and non-availability of on-line or prior-treatment in-vivo verification results. Although the PSQA method reported by Takahashi [12] was carried out in a single irradiation, it requires a customized phantom with many slabs, three ion chambers and three films stitched one after the other to cover 100 cm length of the target. Besides extensive logistic requirements, it is very labor intensive, time consuming and co-registration of film measured and TPS calculated dose fluence is not straight forward and error prone.

The feasibility of using MVCT measured LOTS to reconstruct 3D dose distribution on to patient CT datasets have been reported by several authors either by using in-build or independent dose reconstruction algorithm in routine clinical cases [13–19, 21]. The excellent agreement both in absolute dose (< ± 1.8%), 2Dγ (> 97%), 3Dγ (> 96%) and 2Dγ vs 3Dγ variation (≤ 2.5%) between reconstructed dose from LOTS as compared to planned, ion chamber and ArcCHECK measurement, in all ten non-TMI patients validated the accuracy and reliability of LOTS reconstructed method in Delivery Analysis against the standard methods. Our results are in agreement with previous publications [17–19]. Although MVCT measured LOTS reconstructed 3D dose distribution has been successfully implemented as an alternative PSQA method for regular clinical cases, its feasibility in TMI/TMLI has not been reported so far. The feasibility and validation of this method for TMI/TMLI is especially important as there is no direct approach and suitable detector or phantom to verify the delivery accuracy of this complex and highly modulated mega field.

In a big surprise to our retrospective investigation of LOTS reconstructed 3D dose distribution in five TMI/TMLI patients, 3Dγ to the majority of the PTVs in upper body plans were found much below the acceptable criteria of 95%. Amongst the four separate PTVs (Brain, Chest, Torso and limb), 3Dγ were slightly better for brain (> 90%) except P12. The 3Dγ values (28%-94%) of PTV Chest of every patient were much lesser than corresponding 2Dγ (> 96%) estimated from ArcCHECK measurement. For reasons not clear to us, we observed poor 3Dγ values from the analysis of the first patient (P11) itself. In an attempt to remove any possible error, we carried out a series of investigations including various email communication and data sharing with Accuray Medical Physics support team based in Europe. MVCT detector output was re-calibrated and baselined again following recommended protocol described in the manual. As per the suggestions from Accuray medical physics support, the threshold of LOT was decreased from 0.7 to 0.5. Even after all these probable corrective measures also, the new 3D dose distribution reconstructed for the same patient (P11) from the newly measured LOTS resulted in no changes in the 3Dγ values of all PTVs. Despite this unsatisfactory result, we have continued the measurement of LOTS for the other four patients and reconstructed dose distributions were compared with the corresponding plans. Off the five patients, the upper body TMI/TMLI plan of P14, which was created using 5 cm field width, pitch of 0.3 and modulation factor of 3 showed the best agreement with 3Dγ values ≥ 93% in three separate PTVs. Although 3Dγ values were very poor for all PTVs and for every patient, the median deviation in D_98%_ of all PTVs were within 2.5% except torso where the deviation was 3.78%. The deviation in D_2%_ was relatively large for all PTVs and increase up to 9.48% for lower body PTV where 3Dγ were 100%. Overall, minimum PTV coverage (D_98%_) from the reconstruction method was within ± 5% of corresponding plans except for PTV upper body where a reduction of up to 6.33% was observed. The reconstruction method increases hot spot (D_2%_) to all PTVs by up to 12.51% as compare to plan. The overall analysis results based on minimum and maximum dose to PTVs can still be considered acceptable, although not very satisfactory, based on the complexity of the target and treatment technique.

The selection of optimum field width, pitch and modulation factor determine both TMI/TMLI plan quality and treatment time. Hui et al. [4] have investigated the effect of field width, modulation factor and pitch on the treatment plan outcome and delivery time. The authors have reported a reduction of treatment delivery time by 50% when the field width was increased from 2.5 to 5 cm in superior-inferior direction. For a 5 cm field width, earlier studies have recommended modulation factor and pitch ranging from 2.0 to 2.8 and 0.397 to 0.46 [4, 7, 8] respectively. The impact of HT planning parameters on the PSQA results especially with reconstruction from measured LOTS has not been reported in the literature. In all the lower body TMI/TMLI plans where the 3Dγ values were 100%, a field width of 5 cm, modulation factor from 2.15 to 2.5 and pitch from 0.4 to 0.41 were used, which is in agreement with the reported values [4, 7, 8]. However, in the upper body TMI/TMLI plans, modulation factor and pitch were customized from 2.49 to 3.5 and 0.3 to 0.43 to meet the set clinical goals. Although both modulation factor of 2.49 and 3.5 provides a poor 3Dγ values in patients P11 and P15, we observed a fairly better 3Dγ values of P14 plan created with modulation factor of 3 and pitch of 0.3. Subsequently, all the upper body TMI/TMLI plans including for patient P14 were re-optimized with 5 cm field width, pitch of 0.3 and modulation factor of 3. Moreover, even after getting optimum dose distribution and MLC-LOT, we continue to run up to 1000 iteration, while simultaneously ensuring no change in the plan quality. The reconstructed dose from the new upper body plans thus created showed improvement both in 3Dγ and minimum target coverage and hot spot. As the 3Dγ was 100% for all plans and PTVs, we have tightened the evaluation criteria to 2%@2mm. Even at this stringent evaluation criteria also, almost all plans pass acceptance criteria of 95%. This also leads to improvement in the deviation in D_98%_ and D_2%._ However, only increase of pitch to 0.43 without changing other plan parameters did not resulted any improvement both in 3Dγ, D_98%_ and D_2%_.

Although, all the original plans were deliverable on RadiXact HT and passed the traditional ion chamber and ArcCHECK based PSQA, the used HT plan parameters were not optimum for MLC-LOTS based PSQA methods. We proposed a field width of 5 cm, pitch of 0.3 and modulation factor of 3 as an optimum HT plan parameters for TMI/TMLI patients not only to create the most optimum dose distribution but also for a successful implementation of MLC-LOTS based PSQA methods. Measured LOTS based reconstructed methods provide accurate and efficient verification of TMI/TMLI plan in a single irradiation. Reconstructed 3D dose calculation assumes that there is no change in the patient anatomy and tumor geometry. Moreover, the reconstruction method in Delivery Analysis does not explicitly check for differences between planned and delivered gantry angle, couch position, or treatment field position. Only variations in MLC-LOT are considered when calculating dose differences.

## Conclusion

MLC-LOTS based PSQA is accurate, robust and easy to implement in any busy radiation oncology facility without additional logistics, new phantom and detector. It serves as an effective and efficient method of PSQA for HT treatment plans and best suited for very large target like TMI/TMLI. HT treatment plan parameters, mean and maximum LOT greatly influence the reconstructed 3Dγ, D_98%_ and D_2%._ We proposed a field width of 5 cm, pitch of 0.3 and modulation factor of 3 as an optimum HT plan parameters for TMI/TMLI patients not only to create the most optimum dose distribution but also for a successful implementation of MLC-LOTS based PSQA methods.

## Data Availability

Yes, will be supply when requested.

## Abbreviations

TBI: Total body irradiation
MAC: Myeloablative conditioning
ABMT: Allogeneic bone marrow transplant
TMI: Total marrow irradiation
TMLI: Total marrow and lymphoid irradiation
VMAT: Volumetric modulated arc therapy
HT: Helical tomotherapy
PSQA: Patient specific quality assurance
MVCT: Megavoltage cone beam computed tomography
LOTS: Leaf open time sinogram
TPS: Treatment planning system
FFF: Flattening filter free
MLC: Multi-leaf collimator
IC: Ionization chamber
γ: Gamma
3%@3mm: 3% Dose difference at 3 mm distance-to-agreement
DVH: Dose-volume-histogram
D_98%_: Dose to 98% volume
D_2%_: Dose to 2% volume
PTV: Planning target volume
SD: Standard deviation
